# Spherical Shell Bioprinting to Produce Uniform Spheroids with Controlled Sizes

**DOI:** 10.3390/jfb15110350

**Published:** 2024-11-18

**Authors:** Kuk Hui Son, Dong-Ha Kim, Seunghye Park, Hyun Jae Kim, Mira Park, Seung-Jin Kim, Sang Jin Lee, Keunsun Ahn, Jin Woo Lee

**Affiliations:** 1 Department of Thoracic and Cardiovascular Surgery, Gil Medical Center, College of Medicine, Gachon University, 21, Namdong-daero 774 Beon-gil, Namdong-gu, Incheon 21565, Republic of Korea; dr632@gilhospital.com; 2 Research Institute, Sphebio Co., Ltd., 501-ho, 3, Achasan-ro 11ga-gil, Seongdong-gu, Seoul 04796, Republic of Korea; dhkim@sphebio.com (D.-H.K.); hjkim@sphebio.com (H.J.K.); mrpark@sphebio.com (M.P.); sjkim@sphebio.com (S.-J.K.); 3 Department of Health Sciences and Technology, GAIHST, Gachon University, 155, Gaetbeol-ro, Yeonsu-ku, Incheon 21999, Republic of Korea; sseungh123@naver.com; 4 Wake Forest Institute for Regenerative Medicine, Wake Forest University Health Sciences, Medical Center Boulevard, Winston-Salem, NC 27157, USA; sjlee@wakehealth.edu; 5 Department of Molecular Medicine, College of Medicine, Gachon University, 155, Gaetbeol-ro, Yeonsu-ku, Incheon 21999, Republic of Korea

**Keywords:** spheroid, uniform production, bioprinting, controlled size, shell

## Abstract

Conventional cell spheroid production methods are largely manual, leading to variations in size and shape that compromise consistency and reliability for use in cell-based therapeutic applications. To enhance spheroid production, a spherical shell bioprinting system was implemented, enabling the high-throughput generation of uniform cell spheroids with precisely controlled sizes. The system encapsulates cells within thin alginate hydrogel shells formed through bioprinting and ion crosslinking reactions. Alginate–calcium ion crosslinking created alginate shells that contained gelatin-based bioinks with embedded cells, facilitating spontaneous cell aggregation within the shells and eliminating the need for plastic wells. By adjusting cell concentrations in the alginate–gelatin bioink, we achieved precise control over spheroid size, maintaining a sphericity above 0.94 and size deviations within ±10 µm. This method has been successfully applied to various cell types including cancer cells, fibroblasts, chondrocytes, and epithelial cells, demonstrating its versatility. This scalable approach enhances the reliability of cell therapy and drug screening, offering a robust platform for future biomedical applications.

## 1. Introduction

Tissue and organ damage can lead to the loss of functional cells, driving the development of cell therapies aimed at restoring or regenerating tissues [1,2]. Cell therapy utilizes autologous, allogeneic, or xenogeneic cells that are modified or expanded ex vivo for therapeutic, diagnostic, and preventive purposes. Depending on the type of cells, therapies are categorized into somatic cell therapy and stem cell therapy, with applications such as dendritic cells for cancer treatment, chondrocytes for cartilage repair, and mesenchymal stem cells (MSCs) for tissue regeneration [3,4,5,6,7,8,9].

However, the direct transplantation of therapeutic cells often encounters challenges, including external stress, unintended migration, and low viability, which can reduce therapeutic outcomes [5]. To address these limitations, scaffold-based methods embed cells in hydrogels to provide structural support, while scaffold-free methods, such as spheroid cultures, promote natural cell aggregation and enhance cell–cell communication [10,11,12,13,14]. Spheroids better mimic the physiological microenvironment, making them ideal for cell therapy, drug screening, and disease modeling.

Several techniques have been developed to produce spheroids, including the hanging drop method, liquid overlay, spinner flasks, and microfluidic chips [15,16,17,18,19,20]. However, these methods suffer from limitations such as inconsistent spheroid size, low scalability, and high shear stress, which can damage cells and reduce viability [21,22,23,24].

To address these challenges, 3D bioprinting has emerged as a promising solution for integrating cells and biomaterials into bioinks to create precise 3D structures [25]. Among the various approaches, micro-extrusion-based bioprinting offers fine control over droplet size and patterning, enhancing scalability and reproducibility [26]. Recent studies have demonstrated the potential of bioprinting to improve cell–cell interactions and construct complex multilayered tissues [27,28,29]. However, obtaining uniform spheroids with controlled sizes, high sphericity, and consistency remains challenging.

This study introduces a spherical shell bioprinting (SSB) system that is designed to produce highly uniform spheroids by encapsulating cells within ion-crosslinked alginate hydrogel shells [30]. Hydrogels are synthesized by polymerization via physical and chemical crosslinking pathways. Among them, the use of ionic polymerization for the crosslinking of alginate, that is, one of the chemical crosslinking pathways, has the advantage of being able to create a spherical shell in a short period of time because it has high reaction sensitivity in aqueous environments [31]. The automated system dispenses bioink droplets into spherical shells, where cells aggregate to form spheroids. After maturation, the alginate shells are easily degraded, enabling efficient harvesting. The spheroid size can be precisely controlled by adjusting the cell concentrations in the bioink, and the system is compatible with various cell types, including cancer cells, fibroblasts, chondrocytes, and stem cells. This approach ensures consistent and scalable spheroid production, providing a reliable platform for cell therapy and drug screening.

## 2. Materials and Methods

### 2.1. Materials

Gelatin (CAS: 9000-70-8), sodium alginate (CAS: 9005-38-3), calcium chloride (CAS: 10043-52-4), and alginate lyase (CAS: 9024-15-1) were obtained from Sigma-Aldrich (St. Louis, MO, USA). Needles (14 G, 25 G, and 30 G), Luer-lock syringes (10 mL), and related accessories were purchased from Banseok Precision IND (Seoul, Republic of Korea) and BD Biosciences (Franklin Lakes, NJ, USA). Spheroid grid-well dishes (no. 111350) were supplied by SPL Life Sciences (Gyeonggi-do, Republic of Korea). EDTA (0.5 M) was purchased from Welgene (Gyeongsangbuk-do, Republic of Korea). Cell strainers (40 µm and 1000 µm) were purchased from pluriSelect (Leipzig, Germany).

### 2.2. Spherical Shell Bioprinting (SSB) System

**Figure 1:**
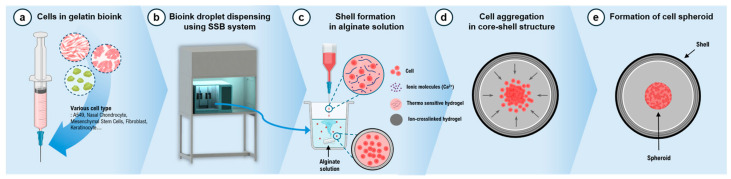
Schematic images of the spheroid production process using the SSB system. (**a**) Preparation of bioink based on a thermo-sensitive hydrogel mixed with cells and ionic molecules. (**b**) Illustration of the SSB system. (**c**) A specialized spherical shell bioprinting system dispenses bioink droplets containing cells and ionic molecules into a sodium alginate solution, creating core–shell structures through an ion-crosslinked hydrogel bound to ionic molecules on the surface of the bioink droplet. (**d**) Cells aggregate in the center of the core–shell structure. (**e**) Cell spheroids are formed as the cell culture progresses.

A novel SSB system was developed for precise spheroid production (Figure 1). The system was installed in a biosafety cabinet to prevent contamination from external components. It featured three temperature-controlled bioink dispensing units, each equipped with multi-head nozzles to enhance printing efficiency. The temperature of the dispensing units could be adjusted between 0 °C and 25 °C, with a controllable dispensing speed ranging from 0 to 99 mL/min. An agitator located at the bottom of each dispensing unit was rotated at speeds between 0 and 500 rpm to facilitate interaction between the bioink droplets and the alginate solution. Under optimized conditions, the bioink was dispensed at 1 mL/min with the agitator rotating at 250 rpm, enabling the production of approximately 400 bioink droplets per minute and an equivalent number of spheroids.

### 2.3. Cell Isolation and Culture

A549 lung carcinoma cells were purchased from ATCC (Manassas, VA, USA) and cultured in Alpha-MEM (Welgene, Republic of Korea) supplemented with 10% fetal bovine serum (FBS; Gibco, Waltham, MA, USA) and 1% penicillin/streptomycin (Welgene). Human normal fibroblast (hNF) cells were obtained from PromoCell (Heidelberg, Germany) and cultured in high-glucose Dulbecco’s modified Eagle’s medium (DMEM; Hyclone, Logan, UT, USA) supplemented with 10% FBS and 1% penicillin/streptomycin. Human mesenchymal stem cells (hMSC), human nasal chondrocytes (hNC), and human adipose-derived stem cells (hADSC) were obtained from Seoul St. Mary’s Hospital, The Catholic University of Korea, and cultured in low-glucose DMEM (Hyclone) supplemented with 10% FBS and 1% penicillin/streptomycin. Human normal keratinocytes (hNK) were obtained from Seoul St. Mary’s Hospital and cultured in KBM-Gold basal media (Lonza, Basel, Switzerland) supplemented with KGM-Gold SingleQuots (Lonza) and 1% penicillin/streptomycin. Culture media were replaced every 2–3 days and subculturing was performed when the cells reached 70–80% confluence.

### 2.4. Spheroid Production Process

#### 2.4.1. Bioink Preparation

The bioink was prepared by mixing a 4% gelatin solution, a 1 M calcium chloride solution, and the cell suspension. For the gelatin solution, 4 g of gelatin powder was dissolved in 100 mL of culture medium containing 1% penicillin/streptomycin and stirred at 40 °C for 24 h. The solution was then sterilized using a 0.45 µm filter and sealed. The calcium chloride solution was prepared by dissolving 4.4 g of calcium chloride in 40 mL of deionized water and filtering it through a 0.22 µm filter. Similarly, 0.4 g of sodium alginate was dissolved in 100 mL of deionized water, filtered through a 0.45 µm filter, and cooled to 4 °C. Cells at appropriate confluence were detached using 0.25% trypsin–EDTA and resuspended in culture medium. The gelatin and calcium chloride solutions were combined with the cell suspension to obtain a final concentration of 2% gelatin and 40 mM calcium chloride. The bioink was mixed thoroughly to ensure even cell distribution, transferred into a 10 mL Luer-lock syringe, and cooled at 4 °C for 45 min.

#### 2.4.2. Production of Spherical Beads and Cell Spheroids

The cooled bioink was dispensed through a 25G needle (inner diameter: 0.28 mm) attached to a syringe mounted on the SSB syringe holder. A 100 mL reservoir containing 0.4% sodium alginate solution at 4 °C was placed under the dispensing head, which was equipped with a stirrer. The bioink was dispensed at 1.0 mL/min and a volume of each droplet was 7.4 µL. As the droplets entered the alginate solution, an ionic crosslinking reaction occurred, forming spherical beads with a core–shell structure (core: 2% gelatin solution with cells; shell: alginate gel). After dispensing all the bioinks, 100 mL of cold sterile distilled water was added to stop the crosslinking reaction. The beads were washed with cold water, transferred to culture dishes, and immersed in culture medium. The cells were incubated at 37 °C with 5% CO_2_ for 72 h to allow for spheroid formation.

#### 2.4.3. Spheroid Harvesting

After the spheroids formed, the beads were removed from the culture medium and washed with Dulbecco’s phosphate-buffered saline (DPBS, Gibco). The beads were then immersed in a 5 mM EDTA solution with gentle stirring to degrade the alginate shell. Once the shell was fully degraded, the spheroids were filtered through a 40 µm cell strainer, washed thoroughly with DPBS, and collected for further analysis.

### 2.5. Spheroid Production Process Using Grid-Well Culture Dish

The grid area at the center of the spheroid dish was sterilized with 70% ethanol and washed thrice with DPBS to remove residual ethanol. The remaining DPBS was removed by vacuum suction to ensure a dry surface. A cell suspension containing 5 × 10^5^ cells in 800 µL of culture medium was evenly spread across the grid area. The dish was incubated at 37 °C with 5% CO_2_ for 72 h. After incubation, spheroid formation was confirmed by microscopy. To collect the spheroids, DPBS was sprayed onto the grid area, and the spheroids were carefully transferred for further analysis.

### 2.6. Phase Microscopy and Image Analysis

All the separated spheroids were imaged using an inverted optical microscope (CKX53; Olympus, Tokyo, Japan). Image scaling was performed using a calibration slide to ensure accurate measurement. The open-source software ImageJ (Fiji package, open-source) was used to measure the diameter, perimeter, and area of the spheroids. Data from ImageJ were further analyzed in Excel, where the measured area (S) from the 2D projection of the spheroids was used to calculate the radius (r) and the volume [V = (4/3)πr^3^] of an equivalent sphere. Additionally, the open-source ReViSP software was used to perform the 3D analyses of spheroid size and shape, including volume and sphericity, and to generate 3D-rendered images.

### 2.7. Cell Viability

Spheroids suspended in DPBS were centrifuged at 1500× *g* rpm for 10 min. After removing the supernatant, the spheroids were stained with 4 µM EthD-1 and 2 µM Calcein AM from the LIVE/DEAD Viability/Cytotoxicity Kit (Invitrogen, Waltham, MA, USA). Samples were incubated in the dark at room temperature for 30 min, washed with DPBS, and observed under a confocal laser scanning microscope (SP8X; Leica, Wetzlar, Germany) and an inverted optical microscope (CKX53; Olympus, Tokyo, Japan). The cell viability of the spheroids was calculated by analyzing the fluorescence images obtained through a confocal microscope using the ImageJ program [32].

### 2.8. Spheroid Aspiration Test

A 5 mL suspension of spheroids, produced using both the grid-well dish and SSB system, was aspirated into a 10 mL syringe fitted with a 30G needle (inner diameter: 0.16 mm). The aspiration test was performed under identical injection pressure (230 kPa) conditions with a dispensing speed of 10 mL/min for each syringe. The entire volume of the ejected suspension was collected and examined under a microscope to assess spheroid morphology and detect dissociated single cells.

### 2.9. Statistical Analysis

Data are presented as the mean ± SD from at least three independent experiments. One-way ANOVA was performed, followed by Tukey’s post hoc test for multiple comparisons. Student’s *t*-test was used for comparisons between two groups and *p* < 0.05 was considered statistically significant.

### 2.10. Nomenclature & Abbreviations

The abbreviations used in the manuscript are summarized in Table 1.

## 3. Results

### 3.1. Spheroid Production Using the SSB System

**Figure 2:**
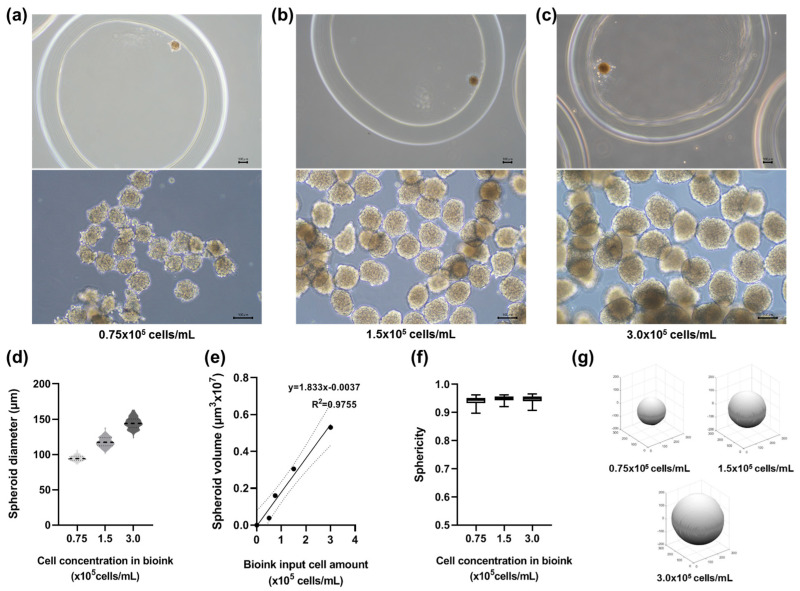
Size-controlled spheroids produced by SSB. (**a**–**c**) Spheroids with diameters of approximately 94 µm, 117 µm, and 143 µm were generated using bioinks containing 0.75 × 10^5^, 1.5 × 10^5^, and 3.0 × 10^5^ cells/mL, respectively (scale bar = 100 µm). (**d**) Box plot of spheroid diameters. Statistical analysis using one-way ANOVA followed by Tukey’s post hoc test shows significant differences (*p* < 0.001). (**e**) Linear correlation between spheroid volume and cell number (R^2^ = 0.98), confirming precise size control. (**f**) All spheroids maintained sphericity values above 0.94, ensuring structural integrity (*p* < 0.05). (**g**) 3D renderings of representative spheroids illustrate their consistent morphology across different concentrations.

Spheroids were produced using the SSB system. Gelatin-based bioinks with three different hNC cell concentrations (0.75 × 10^5^, 1.5 × 10^5^, and 3.0 × 10^5^ cells per mL) were prepared and dispensed into a 0.4% sodium alginate solution to create beads with an alginate shell (thickness: 200 µm) (Figure 2a–c). After the alginate shell was degraded with alginate lyase, spheroids with diameters of 95.3 ± 6.1 µm, 117.7 ± 8.7 µm, and 143 ± 8.7 µm were obtained, depending on the initial cell concentrations (Figure 2d). A linear relationship was observed between the spheroid volume and the number of encapsulated cells (Figure 2e). Additionally, all spheroids maintained a sphericity greater than 0.94, confirming their near-spherical structures (Figure 2f). The SSB system produced approximately 400 spheroids per minute, representing a significant improvement in throughput compared with conventional grid-well methods.

### 3.2. Cell Viability in SSB-Based Spheroids

**Figure 3:**
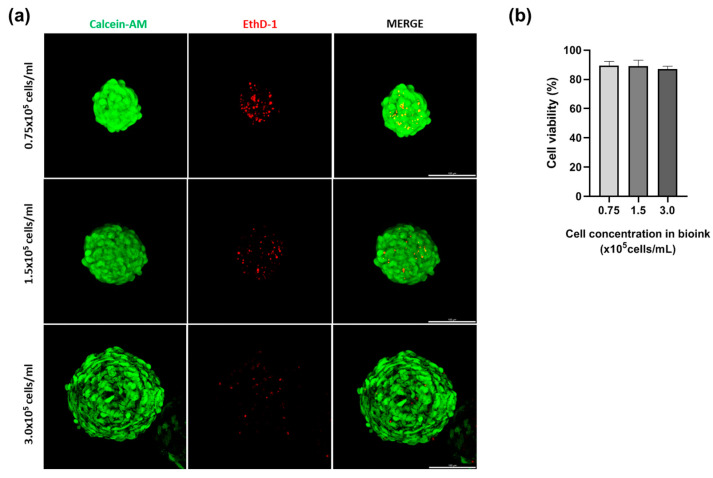
Cell viability of spheroids produced using the SSB system. (**a**) Confocal microscopy images display live (green, Calcein-AM) and dead (red, EthD-1) cells within spheroids (scale bar = 100 µm). (**b**) Quantitative analysis shows that all spheroids maintained > 85% viability, with higher densities (3.0 × 10^5^ cells/mL) resulting in improved structural stability (*p* < 0.05). These results highlight the system’s suitability for producing robust spheroids for therapeutic applications.

The viability of spheroids produced with different cell densities (0.75 × 10^5^, 1.5 × 10^5^, and 3.0 × 10^5^ cells per mL) was assessed using live and dead staining. All spheroids exhibited a viability rate greater than 85% (Figure 3). Although 0.75 × 10^5^ cells per mL spheroids showed the best cell viability, the *p* value in the three concentration conditions was 0.61, so the difference was not significant. The spheroids produced at higher cell concentrations showed improved aggregation, resulting in more stable spherical structures. These results demonstrate the potential of SSB-generated spheroids for applications requiring robust and functional cell constructs, such as cell therapy.

### 3.3. Comparison Between Grid-Well Culture Dish and SSB

**Figure 4:**
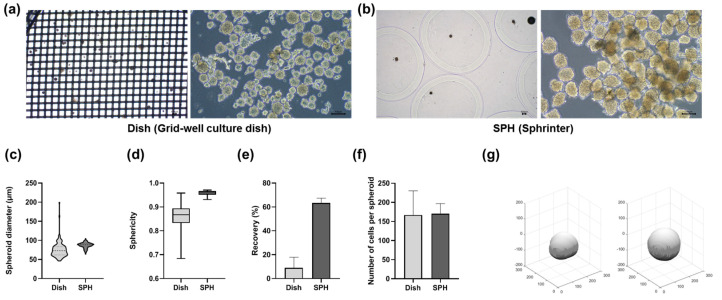
Comparison between grid-well dish and SSB spheroids. (**a**) Spheroids from the grid-well dish show irregular diameters and inconsistent morphology (scale bar = 100 µm). (**b**) The SSB system produced spheroids with uniform size and shape (scale bar = 100 µm). (**c**) Box plot comparison of spheroid diameters shows significantly reduced variability with the SSB system (*p* < 0.001). (**d**) Sphericity analysis reveals better uniformity in SSB spheroids (0.95 ± 0.02) compared to grid-well spheroids (0.86 ± 0.08, *p* < 0.05). (**e**) The SSB system achieved a higher spheroid conversion rate (63.3%) compared to that by the grid-well dish (8.9%, *p* < 0.001), demonstrating improved efficiency. (**f**) No significant difference was found in the number of cells per spheroid between the two methods (grid-well: 167 ± 52; SSB: 170 ± 22, n.s.). (**g**) 3D renderings highlight the morphological differences between spheroids produced by each method.

To evaluate the performance of the developed technology, the spheroids generated using a commercial grid-well dish were compared with those produced using the SSB system. Spheroids from the grid-well dish exhibited inconsistent diameters, whereas the SSB system consistently produced spheroids of uniform size (Figure 4a,b). Although the average diameters were similar (75 ± 25 µm for the grid-well dish and 89 ± 8 µm for the SSB), the variability in diameter was significantly greater for the grid well dish (Figure 4c,g). The spheroids produced by the SSB system exhibited a near-perfect spherical structure with a sphericity value of 0.95 ± 0.02, while those from the grid-well dish had a lower sphericity of 0.86 ± 0.08 (Figure 4d). The spheroid conversion rate, defined as the proportion of cells that successfully aggregated into spheroids, was significantly higher in the SSB system (63.3%) than that in the grid-well dish (8.9%; Figure 4e). The average number of cells per spheroid was comparable between the two methods, with 167 ± 52 cells for the grid-well dish and 170 ± 22 cells for the SSB system (Figure 4f,g). However, fragmented cell clusters were observed at the bottom of the grid-well dish, indicating incomplete aggregation and suggesting that not all cells participated in spheroid formation. These findings highlight the advantages of the SSB system in producing uniform and compact spheroids with minimal size and shape variability, ensuring high consistency and efficiency. Reliability is crucial for applications in drug screening, disease modeling, and cell therapy, where reproducibility and uniformity are essential for success.

### 3.4. Verification of Spheroid Production in Various Cells

**Figure 5:**
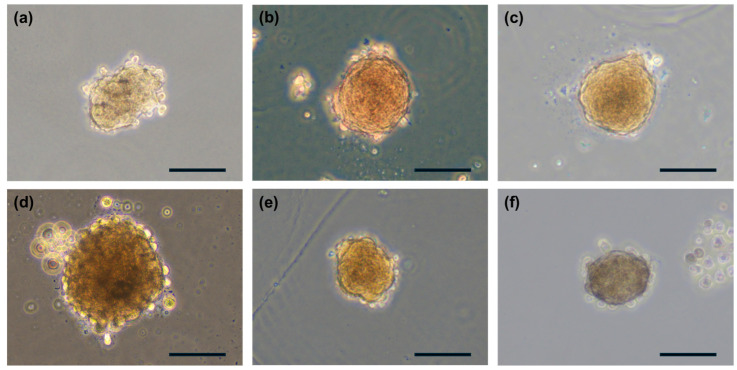
Spheroid formation using various cell types with the SSB system. (**a**–**f**) Spheroids were successfully generated from A549 cancer cells, hNF, hNC, hMSC, hADSC, and hNK (scale bar = 100 µm).

Spheroids were generated from various cell types to validate the applicability of the developed spheroid-production technology. These included cancer cells (A549), stem cells such as hMSC and hADSC, and normal cells, including hNC, hNK, and hNF (Figure 5). The results confirmed that spheroids could be consistently produced across all cell types, with diameters maintained at approximately 100 µm. The ability to generate uniform spheroids from diverse cell types demonstrates the flexibility and versatility of the SSB system. This consistency is critical for applications in cancer research, tissue engineering, drug screening, and other biomedical fields.

### 3.5. Stability of Fabricated Spheroids

**Figure 6:**
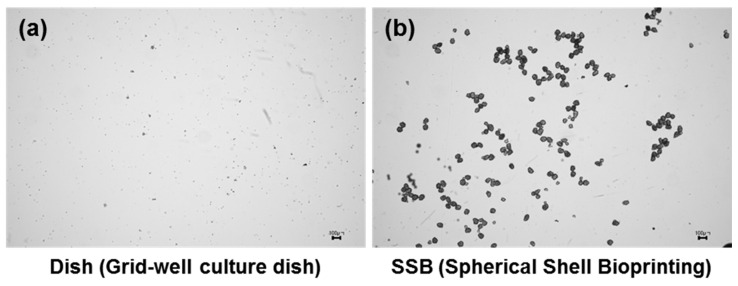
Stability of spheroids during injection through a 30G needle. (**a**) Spheroids from the grid-well dish exhibited size variations and structural damage after injection (scale bar = 100 µm). (**b**) In contrast, spheroids generated using the SSB system maintained their integrity, showing no dissociation into single cells during injection. This result underscores the importance of size uniformity and mechanical stability for therapeutic applications.

To effectively utilize the spheroids produced in cell therapy, their structural stability during injection is critical. In an aspiration test using a 30G needle (inner diameter: 160 µm), spheroids generated by the SSB system at a concentration of 5.0 × 10^4^ cells per mL (average size: 89.1 µm) maintained their integrity and showed no dissociation into single cells. In contrast, spheroids produced using a commercial grid-well dish exhibited size variability, structural damage, and breakage during injection (Figure 6). These findings emphasize the importance of generating spheroids with consistent sizes and robust structures for use in cell therapy. Uniform spheroids reduce the risk of mechanical damage during injection and ensure reliable therapeutic outcomes, highlighting the superiority of the SSB system for clinical applications.

## 4. Discussion

The limitations of 2D cell cultures in replicating in vivo environments have driven the development of 3D cultures that better support cell–cell interactions and tissue formation. Bioprinting technologies have emerged as essential tools for constructing complex tissues by embedding cells within hydrogels. However, traditional bioprinting methods that utilize individual cells often suffer from low cell density and limited functionality, rendering them inadequate for mimicking native tissues [33,34,35,36]. Spheroid-based bioprinting addresses these challenges by promoting natural cell aggregation into 3D structures and enhancing cell signaling and tissue formation [37].

Several bioprinting strategies have been developed to improve the production and application of spheroids, such as extrusion-based [38,39,40,41], droplet-based [42,43], Kenzan method [44,45,46,47], biogripper [48,49], aspiration-assisted [28,50,51], and magnetic bioprinting [52,53]. However, these methods often encounter challenges such as shear stress-induced damage, nozzle clogging, and low throughput, which compromise reproducibility and stability. Given that human organs have high cell densities of 1–3 billion cells/mL, high-throughput spheroid production is essential for tissue engineering and cell-based therapies [54].

The SSB system presented in this study offers a novel solution by producing highly uniform spheroids with consistent diameters of approximately 100 μm. Its core–shell structure enables precise control over size and facilitates easy spheroid retrieval after alginate shell degradation. This system eliminates the need for cell-repellent U-bottom plates, thereby reducing production costs and increasing efficiency. Compared to grid-well dishes, the SSB system achieved higher uniformity and sphericity (above 0.94), reducing the risk of shear stress and prevented blockage during the printing processes [37].

An aspiration test further confirmed the suitability of the SSB system for clinical applications. SSB-based spheroids maintained their integrity during injection through a 30 G needle, whereas spheroids from grid-well dishes exhibited size variations, fragmentation, and instability, compromising their utility in cell therapies. These findings demonstrate the importance of size consistency and mechanical stability for therapeutic applications. In addition, the spheroids produced in this experiment were confirmed to have maintained their shape and cell survival up to 7 days and to have shown a high survival rate after 6 months of storage in liquid nitrogen. However, further research on long-term survival and storage methods will be necessary for actual clinical use.

Additionally, the SSB system demonstrated versatility by producing spheroids from a variety of cell types, including cancer, stem, and normal human cells. This flexibility makes these spheroids suitable for drug screening, disease modeling, and tissue engineering [55,56,57]. With increasing restrictions on animal models in preclinical research, 3D spheroid cultures have become essential tools for studying complex biological processes [58]. Moreover, the integration of patient-derived cells, such as induced pluripotent stem cells, into spheroid bioprinting offers exciting opportunities for personalized therapies and advancements in regenerative medicine [59].

The SSB system bridges the gap between laboratory research and clinical application by offering a scalable, efficient, and versatile platform. Its ability to produce high-throughput uniform spheroids supports their diverse applications in bioprinting-based therapies, drug screening, and tissue engineering.

## 5. Conclusions

This study introduced the SSB system, a novel spherical shell bioprinting platform that is capable of producing highly uniform spheroids with precise control over their size and sphericity. The system generated spheroids with consistent diameters of approximately 100 µm across various cell types, including cancer cells, stem cells, and normal cells, demonstrating its versatility and reproducibility. Compared to conventional methods, such as grid-well dishes, the SSB system achieved greater size uniformity, sphericity, and mechanical stability. In aspiration tests simulating clinical injections, the SSB-based spheroids maintained their integrity without dissociation, confirming their potential for use in cell therapy. The scalability and flexibility of SSB systems offer significant advantages for tissue engineering, disease modeling, and drug screening. Future studies should focus on co-culture models and patient-derived cells to replicate complex tissue structures and validate the utility of this system in personalized medicine. Owing to its robust design and efficient performance, our system presents a valuable platform for advancing regenerative medicine and bioprinting-based innovations in both research and clinical applications.

## Figures and Tables

**Table 1:** Abbreviation.

Abbreviation	Full Name
MSCs	mesenchymal stem cells
2D	two-dimensional
3D	three-dimensional
SSB	spherical shell bioprinting
gelatin	gelatin from bovine skin
CaCl_2_	calcium chloride
EDTA	ethylenediaminetetraacetic acid
A549	human lung cancer cells
hNFs	human normal fibroblasts
DMEM	Dulbecco’s modified Eagle’s medium
FBS	fetal bovine serum
P/S	penicillin/streptomycin
Alpha-MEM	alpha-minimum essential medium
hNCs	human nasal chondrocytes
hADSCs	human adipose-derived stem cells
hNKs	human normal keratinocytes
KBM	keratinocyte basal medium
KGM	keratinocyte growth medium
DPBS	Dulbecco’s phosphate-buffered saline
calcein-AM	calcein-acetoxymethyl
EthD-1	ethidium homodimer-1
G	gauge

## Data Availability

The original contributions presented in this study are included in the article. Further inquiries can be directed to the corresponding authors.

## References

[1] El-Kadiry A.E., Rafei M., Shammaa R. (2021). Cell Therapy: Types, regulation, and clinical benefits. Front. Med..

[2] Kim I. (2013). A brief overview of cell therapy and its product. J. Korean Assoc. Oral Maxillofac. Surg..

[3] Golchin A., Farahany T.Z. (2019). Biological products: Cellular therapy and FDA approved products. Stem Cell Rev. Rep..

[4] Mount N.M., Ward S.J., Kefalas P., Hyllner J. (2015). Cell-based therapy technology classifications and translational challenges. Philos. Trans. R Soc. B Biol. Sci..

[5] De Pieri A., Rochev Y., Zeugolis D.I. (2021). Scaffold-free cell-based tissue engineering therapies: Advances, shortfalls and forecast. NPJ Regen. Med..

[6] Sánchez A., Schimmang T., García-Sancho J. (2012). Cell and tissue therapy in regenerative medicine. Adv. Exp. Med. Biol..

[7] Gurusamy N., Alsayari A., Rajasingh S., Rajasingh J. (2018). Adult stem cells for regenerative therapy. Prog. Mol. Biol. Transl. Sci..

[8] Takahashi K., Tanabe K., Ohnuki M., Narita M., Ichisaka T., Tomoda K., Yamanaka S. (2007). Induction of pluripotent stem cells from adult human fibroblasts by defined factors. Cell.

[9] Schwartz S.D., Hubschman J.P., Heilwell G., Franco-Cardenas V., Pan C.K., Ostrick R.M., Mickunas E., Gay R., Klimanskaya I., Lanza R. (2012). Embryonic stem cell trials for macular degeneration: A preliminary report. Lancet.

[10] Hollister S.J. (2005). Porous scaffold design for tissue engineering. Nat. Mater..

[11] Nichol J.W., Khademhosseini A. (2009). Modular tissue engineering: Engineering biological tissues from the bottom up. Soft Matter..

[12] Yamato M., Okano T. (2004). Cell sheet engineering. Mater. Today.

[13] Matsuda N., Shimizu T., Yamato M., Okano T. (2007). Tissue engineering based on cell sheet technology. Adv. Mater..

[14] Haraguchi Y., Shimizu T., Sasagawa T., Sekine H., Sakaguchi K., Kikuchi T., Sekine W., Sekiya S., Yamato M., Umezu M. (2012). Fabrication of functional three-dimensional tissues by stacking cell sheets in vitro. Nat. Protoc..

[15] Lee D.H., Yun D.W., Kim Y.H., Im G.B., Hyun J., Park H.S., Bhang S.H., Choi S.H. (2023). Various three-dimensional culture methods and cell types for exosome production. Tissue Eng. Regen. Med..

[16] Kim M., Yun H.W., Park D.Y., Choi B.H., Min B.H. (2018). Three-dimensional spheroid culture increases exosome secretion from mesenchymal stem cells. Tissue Eng. Regen. Med..

[17] Costa E.C., Gaspar V.M., Coutinho P., Correia I.J. (2014). Optimization of liquid overlay technique to formulate heterogenic 3D co-cultures models. Biotechnol. Bioeng..

[18] Chaicharoenaudomrung N., Kunhorm P., Noisa P. (2019). Three-dimensional cell culture systems as an in vitro platform for cancer and stem cell modeling. World J. Stem Cells.

[19] Bauer S., Huldt C.W., Kanebratt K.P., Durieux I., Gunne D., Andersson S., Ewart L., Haynes W.G., Maschmeyer I., Winter A. (2017). Functional coupling of human pancreatic islets and liver spheroids on-a-chip: Towards a novel human ex vivo type 2 diabetes model. Sci. Rep..

[20] Manzoor A.A., Romita L., Hwang D.K. (2021). A review on microwell and microfluidic geometric array fabrication techniques and its potential applications in cellular studies. Can. J. Chem. Eng..

[21] Mattot V., Raes M.B., Henriet P., Eeckhout Y., Stehelin D., Vandenbunder B., Desbiens X. (1995). Expression of interstitial collagenase is restricted to skeletal tissue during mouse embryogenesis. J. Cell Sci..

[22] Foglietta F., Canaparo R., Muccioli G., Terreno E., Serpe L. (2020). Methodological aspects and pharmacological applications of three-dimensional cancer cell cultures and organoids. Life Sci..

[23] Moshksayan K., Kashaninejad N., Warkiani M.E., Lock J.G., Moghadas H., Firoozabadi B., Saidi M.S., Nguyen N.T. (2018). 2018 Spheroids-on-a-chip: Recent advances and design considerations in microfluidic platforms for spheroid formation and culture. Sens. Actuators B.

[24] Ho V.H.B., Müller K.H., Barcza A., Chen R., Slater N.K.H. (2010). Generation and manipulation of magnetic multicellular spheroids. Biomaterials.

[25] Ahn C.B., Lee J.H., Kim J.H., Kim T.H., Jun H.S., Son K.H., Lee J.W. (2022). Development of a 3D subcutaneous construct containing insulin-producing beta cells to treat type I diabetes. Bio-Des. Manuf..

[26] Jeon S., Heo J.H., Kim M.K., Jeong W., Kang H.W. (2020). High-precision 3D bio-dot printing to Improve paracrine interaction between multiple types of cell spheroids. Adv. Funct. Mater..

[27] Park Y., Ji S.T., Yong U., Das S., Jang W.B., Ahn G., Kwon S.M., Jang J. (2021). 3D bioprinted tissue-specific spheroidal multicellular microarchitectures for advanced cell therapy. Biofabrication.

[28] Ayan B., Wu Y., Karuppagounder V., Kamal F., Ozbolat I.T. (2020). Aspiration-assisted bioprinting of the osteochondral interface. Sci. Rep..

[29] Barcena A.J.R., Dhal K., Patel P., Ravi P., Kundu S., Tappa K. (2023). Current Biomedical Applications of 3D-Printed Hydrogels. Gels.

[30] Nele V., Wojciechowski J.P., Armstrong J.P.K., Stevens M.M. (2020). Tailoring gelation mechanisms for advanced hydrogel applications. Adv. Funct. Mater..

[31] Maitra J., Shukla V.K. (2014). Cross-linking in hydrogels—A review. Am. J. Polym. Sci..

[32] Neto A.I., Correia C.R., Oliveira M.B., Rial-Hermida M.I., Alvarez-Lorenzo C., Reis R.L., Mano J.F. (2015). A novel hanging spherical drop system for the generation of cellular spheroids and high throughput combinatorial drug screening. Biomater. Sci..

[33] Sun W., Starly B., Daly A.C., Burdick J.A., Groll J., Skeldon G., Shu W., Sakai Y., Shinohara M., Nishikawa M. (2020). The bioprinting roadmap. Biofabrication.

[34] Dey M., Ozbolat I.T. (2020). 3D bioprinting of cells, tissues and organs. Sci. Rep..

[35] Ozbolat I.T., Peng W., Ozbolat V. (2015). Application areas of 3D bioprinting. Drug Discov. Today.

[36] Ozbolat I.T. (2015). Bioprinting scale-up tissue and organ constructs for transplantation. Trends Biotechnol..

[37] Banerjee D., Singh Y.P., Datta P., Ozbolat V., O’Donnell A., Yeo M., Ozbolat I.T. (2022). Strategies for 3D bioprinting of spheroids: A comprehensive review. Biomaterials.

[38] Bulanova E.A., Koudan E.V., Degosserie J., Heymans C., Das Pereira F., Parfenov V.A., Sun Y., Wang Q., Akhmedova S.A., Sviridova I.K. (2017). Bioprinting of a functional vascularized mouse thyroid gland construct. Biofabrication.

[39] Mironov V., Visconti R.P., Kasyanov V., Forgacs G., Drake C.J., Markwald R.R. (2009). Organ printing: Tissue spheroids as building blocks. Biomaterials.

[40] Kachurin A., Church K.H., Park H., Mironov V., Markwald R., Vunjak-Novakovic G., Forgacs G. (2008). Tissue engineering by self-assembly of cells printed into topologically defined structures. Tissue Eng..

[41] Mekhileri N.V., Lim K.S., Brown G.C.J., Mutreja I., Schon B.S., Hooper G.J., Woodfield T.B.F. (2018). Automated 3D bioassembly of micro-tissues for biofabrication of hybrid tissue engineered constructs. Biofabrication.

[42] Gutzweiler L., Kartmann S., Troendle K., Benning L., Finkenzeller G., Zengerle R., Koltay P., Stark G.B., Zimmermann S. (2017). Large scale production and controlled deposition of single HUVEC spheroids for bioprinting applications. Biofabrication.

[43] Chen K., Jiang E., Wei X., Xia Y., Wu Z., Gong Z., Shang Z., Guo S. (2021). The acoustic droplet printing of functional tumor microenvironments. Lab Chip.

[44] Moldovan N.I., Hibino N., Nakayama K. (2017). Principles of the kenzan method for robotic cell spheroid-based three-dimensional bioprinting. Tissue Eng. B Rev..

[45] Mitsuzawa S., Ikeguchi R., Aoyama T., Takeuchi H., Yurie H., Oda H., Ohta S., Ushimaru M., Ito T., Tanaka M. (2019). Efficacy of a scaffold-free bio 3D conduit developed from autologous dermal fibroblasts on peripheral nerve regeneration in a canine ulnar nerve injury model: A preclinical proof-of-concept study. Cell Transplant..

[46] Zhang X.-Y., Yanagi Y., Sheng Z., Nagata K., Nakayama K., Taguchi T. (2018). Regeneration of diaphragm with bio-3D cellular patch. Biomaterials.

[47] Takeoka Y., Matsumoto K., Taniguchi D., Tsuchiya T., Machino R., Moriyama M., Oyama S., Tetsuo T., Taura Y., Takagi K. (2019). Regeneration of esophagus using a scaffold-free biomimetic structure created with bio-three-dimensional printing. PLoS ONE.

[48] Ip B.C., Cui F., Tripathi A., Morgan J.R. (2015). The bio-gripper: A fluid-driven micromanipulator of living tissue constructs for additive bio-manufacturing. Biofabrication.

[49] Cui F.R., Ip B.C., Morgan J.R., Tripathi A. (2018). Hydrodynamics of the bio-gripper: A fluid-driven “claw machine” for soft microtissue translocation. SLAS Technol. Transl. Life Sci. Innov..

[50] Ayan B., Heo D.N., Zhang Z., Dey M., Povilianskas A., Drapaca C., Ozbolat I.T. (2020). Aspiration-assisted bioprinting for precise positioning of biologics. Sci. Adv..

[51] Ayan B., Celik N., Zhang Z., Zhou K., Kim M.H., Banerjee D., Wu Y., Costanzo F., Ozbolat I.T. (2020). Aspiration-assisted freeform bioprinting of pre-fabricated tissue spheroids in a yield-stress gel. Commun. Phys..

[52] Ho V.H.B., Guo W.M., Huang C.L., Ho S.F., Chaw S.Y., Tan E.Y., Ng K.W., Loo J.S.C. (2013). Manipulating magnetic 3D spheroids in hanging drops for applications in tissue engineering and drug screening. Adv. Healthc. Mater..

[53] Olsen T.R., Mattix B., Casco M., Herbst A., Williams C., Tarasidis A., Simionescu D., Visconti R.P., Alexis F. (2015). Manipulation of cellular spheroid composition and the effects on vascular tissue fusion. Acta Biomater..

[54] McClelland R.E., Dennis R., Reid L.M., Stegemann J.P., Palsson B., Macdonald J.M., Bronzino E. (2012). Tissue Engineering, Introduction to Biomedical Engineering.

[55] Ingram M., Techy G.B., Saroufeem R., Yazan O., Narayan K.S., Goodwin T.J., Spaulding G.F. (1997). Three-dimensional growth patterns of various human tumor cell lines in simulated microgravity of a NASA bioreactor. Vitro Cell Dev. Biol..

[56] Weiswald L.B., Bellet D., Dangles-Marie V. (2015). Spherical cancer models in tumor biology. Neoplasia.

[57] Napolitano A.P., Dean D.M., Man A.J., Youssef J., Ho D.N., Rago A.P., Lech M.P., Morgan J.R. (2007). Scaffold-free three-dimensional cell culture utilizing micromolded nonadhesive hydrogels. Biotechniques.

[58] Fennema E., Rivron N., Rouwkema J., van Blitterswijk C., De Boer J. (2013). Spheroid culture as a tool for creating 3D complex tissues. Trends Biotechnol..

[59] Rawal P., Tripathi D.M., Ramakrishna S., Kaur S. (2021). Prospects for 3D bioprinting of organoids. Bio-Des. Manuf..

